# Physical exercise improved the hematological effect of vitamin D in type 2 diabetes mellitus-induced nephrotoxicity in rats

**DOI:** 10.1016/j.bbrep.2024.101839

**Published:** 2024-10-04

**Authors:** Halimat Amin Abdulrahim, Adeyemi Fatai Odetayo, Adeoye Tunwagun David, Yusuf Funsho Abdulquadri, Rofiat Oluwasheun Sheu, Pelumi Kikelomo Oluwafemi, Kazeem Bidemi Okesina, Luqman Aribidesi Olayaki

**Affiliations:** a Department of Medical Biochemistry, University of Ilorin, Ilorin, Nigeria; b Department of Physiology, Federal University of Health Sciences, Ila-Orangun, Nigeria; c Department of Biochemistry, Ladoke Akintola University of Technology, Ogbomosho, Nigeria; d Department of Physiology, University of Ilorin, Ilorin, Nigeria; e Department of Physiology, University of Rwanda, Kigala, Rwanda

**Keywords:** Diabetes mellitus, Diabetic kidney disease, renal dysfunction, Nephrotoxicity, Anemia

## Abstract

**Introduction:**

Globally, one of the major causes of renal dysfunction is diabetes mellitus (DM), and diabetic-induced nephrotoxicity has been linked with anemia. Presently, numerous antidiabetic drugs have been designed for the management of this disorder but they possess their undesirable effects such as anemia and acute kidney injury. Hence, we explore the use of vitamin D with or without exercise for the management of DM-induced renal dysfunction.

**Methods:**

Thirty-six (36) Wistar rats were randomly separated into six (6) groups: control (vehicle treated), diabetes untreated (HFD + STZ), diabetes + vitamin D (HFD + STZ + vitamin D), diabetes + exercise (HFD + STZ + exercise), diabetes + vitamin D + exercise (HFD + STZ + vitamin D+ exercise), diabetes + metformin (HFD + STZ + metformin).

**Results:**

Vitamin D with or without exercise significantly reduced T2DM-induced hyperglycemia. Also, a decrease in T2DM-induced increase in urea, creatinine, lactate dehydrogenase, lactate, cholesterol, and triglyceride and a rise in DM-associated reduction in high-density lipoprotein. These events were associated with a significant increase in red blood cells, hematocrit value, hemoglobin, erythropoietin, and a decrease in white blood cell count. Furthermore, vitamin D with or without exercise reversed T2DM-induced increase in pro-oxidant and pro-inflammatory markers. This observed oxido-inflammatory response was associated with a significant increase in xanthine oxidase activities and uric acid concentration. Interestingly, better recovery rates from DM-associated hematological imbalance were discovered in rats co-treated with vitamin D and exercise.

**Conclusion:**

Our findings revealed that exercise enhanced the hematological effect of vitamin D in HFD + STZ-induced T2DM animals.

## Highlights

• T2DM impairs renal functions.
• This is associated with hematological imbalance.
• EX enhanced the ameliorative effect of vitamin D.

## 1 Introduction

Sustained hyperglycemia, as observed in type 2 diabetes mellitus (T2DM), has been associated with cardiovascular disorders resulting from hematological alterations such as lowered hemoglobin concentration (Hb), hematocrit levels or packed cell volume (PCV), and red blood cell (RBC) count [1]; [2]. These alterations have been linked with hypo-regenerative anemia [3] as a result of inadequate erythropoietin synthesis (the hormone majorly produced from the kidney to stimulate RBC production). The fact that the observed hematological alterations in patients with T2DM-induced nephrotoxicity were found to be associated with a decrease in erythropoietin production [4]; [5] further substantiates the importance of kidneys in maintaining hematological status.

With hyperglycemia as the major contributing factor, diabetic nephrotoxicity is considered a major complication of T2DM and the primary factor causing end-stage kidney disease [6]. T2DM is a progressive disease, and its pathogenesis is influenced by cytokines production and oxidative stress-induced poor glycemic control [7]. Also, it has been established that uncontrolled hyperglycemia can activate advanced glycation end-products, which might activate Nuclear factor kappa B (NF-κB) by inhibiting nuclear factor erythroid-derived 2-like2 (Nrf2) (Kaffle et al., 2012). Nrf2 is a regulator of endogenous antioxidants such as catalase (CAT), superoxide dismutase (SOD), and glutathione (GSH) [8], and its inhibition leads to an imbalance between pro-oxidants and antioxidants, thereby leading to oxidative stress. Apart from its antioxidant properties, Nrf2 also performs anti-inflammatory functions by inhibiting the activities of NF-κB [9]. NF-κB stimulates the nucleus via the transcription and release of cytokines (such as interleukins and tumor necrosis factors) to induce an inflammatory response, a significant cause of kidney dysfunction [10].

Since sustained hyperglycemia is the primary cause of T2DM-impaired hematological status, treatment should be aimed at lowering blood glucose. However, most of the established antidiabetic drugs have undesirable effects, e.g., prolonged use of metformin can lead to anemia by causing vitamin B12 malabsorption [11], acute kidney injury [12]. Based on the notable side effects of these conventional drugs, alternative approaches such as diet and lifestyle modification were introduced for managing T2DM-associated complications.

Vitamin D is a hormone that can be obtained from diet, supplements, and sunlight. Vitamin D can be classified into vitamin D2 and D3. Vitamin D2 is obtained from ergosterol and found in yeast, sun dried and ultraviolet irradiated mushrooms, and plants, while vitamin D3 is obtained endogenously from 7-dehydrocholesterol in the skin and found naturally in cod liver oil and oily fish. Once in the circulation, vitamin D is converted by the kidney into the active form (1,25-dihydroxyvitamin D [1,25(OH)2D), which exerts its physiologic functions in the target tissue by binding to the vitamin D receptor (VDR) in the nucleus [13]. Vitamin D is classically known to regulate calcium and phosphate metabolism, however, other physiologic functions have been identified. Vitamin D intake positively correlates with increased hemoglobin levels [14]. Additionally, it has been established that vitamin D directly increases erythropoietin sensitivity in people suffering from kidney dysfunction [15], and lack of vitamin D is associated with hyperparathyroidism, which leads to a decrease in endogenous erythropoietin production [16].

Similarly, exercise has been shown to improve circulatory hematopoietic stem and progenitor cells (HSC/PCs) [17]. In fact, a higher level of HSC/PCs was observed in individuals performing regular exercise compared to their counterparts living sedentary lifestyle. Additionally, exercise improves hematological status by stimulating the synthesis and release of erythropoietin in the kidney [18]. Despite these exciting activities of vitamin D and physical exercise on hematological status, no study has explored the effect of vitamin D and/or exercise in diabetic patients. Hence, we designed this study to uncover the ameliorative effect of vitamin D with or without exercise on T2DM-impaired hematological status and the possible associated mechanism of action.

## 2 Materials and methods

### 2.1 Chemicals

Vitamin D3 was purchased from Principle Healthcare International Limited in the United Kingdom, while metformin was obtained from Osworth Nigeria Limited in Lagos, Nigeria. Also, STZ was acquired from Sigma-Aldrich in St. Louis, Missouri, the United States.

### Ethical approval

The animals used in this study were humanely catered for according to the National Institute of Health (NIH), and experimental findings were reported based on ARRIVE guidelines. Also, the guidelines stated by the National Research Council's for the Care and Use of Laboratory Animals were strictly followed and the study was approved by the institutional ethical review committee with approval no: UERC/ASN/2023/2582.

### 2.2 Experimental procedure

Male Wistar rats (n = 36) of 120–150 g were purchased from the University of Ilorin. The animals were housed and allowed to acclimatize in a conducive environment for one week. After acclimatization, they were randomized into six groups; control (corn oil), diabetes untreated (HFD + STZ), diabetes + 1000 IU/kg of vitamin D (HFD + STZ + vitamin D), diabetes + exercise (HFD + STZ + exercise), diabetes + vitamin D + exercise (HFD + STZ + vitamin D+ exercise), diabetes + 180 mg/kg metformin (HFD + STZ + metformin). All the treatments were via oral gavage and lasted for 28 days except otherwise stated. The choice of vitamin D dosage was chosen based on Expert Group on Vitamins and Minerals [19] recommendations and it is similar to the dosage used and reported by Krisnamurti et al. [20] and Gázquez et al. [21]. Additionally, metformin dosage and exercise period was based on our previous study [22].

### 2.3 Exercise procedure

The animals were trained by having them run on a modified exercise wheel for 5 min a day, at a speed of 20 m/min, for two weeks prior to the start of the experiment (i.e., diabetes induction). After the experiment, the animals in the exercise groups continued to exercise for a further 28 days. The physical activity intensity was comparable to that of Luo et al. [23] and [26] and is within the established intensity range for moderate exercise.

### 2.4 Induction of T2DM

As described by Okesina et al. [25] “the rats were fed a HFD (maize = 5.5 kg, wheat = 0.5 kg, ground nut cake = 5.5 kg, soya meal/cake/full fat = 12.5 kg, palm kernel cake = 5.0 kg, bone meal = 0.5 kg, methionine = 0.25, lysine = 0.25) for 5 weeks”, and after an overnight fasting, they received a single intraperitoneal dosage of STZ (35 mg/kg b.w.) dissolved in a cold citrate buffer [26]. After 72 h of STZ injection, a glucometer (Fine test) was used to measure the rats' blood glucose and rats with blood glucose level above 250 mg/dl were classified as diabetic. Additionally, the control group were received 0.5 mL of citrate buffer via the same route.

### 2.5 Collection of samples

After 12 h from the last dosage, the animals were euthanized via an intraperitoneal injection of 40 and 4 mg/kg of ketamine and xylazine respectively [27]. Blood samples were obtained through cardiac puncture into heparinized bottles, and plasma was obtained via centrifugation at a revolution of 5000 rpm for 15 min. Furthermore, the left kidneys were collected into sufficient amount of phosphate buffer and homogenized (with dilution factor of 1:5) for biochemical assays. The right kidney was also obtained for histological processing.

### 2.6 Biochemical assays

#### 2.6.1 Fasting blood glucose

Fasting blood glucose was estimated by glucose oxidase method using a digital glucometer (On Call®Plus ACON Laboratories, Inc. San Diego, CA).

#### 2.6.2 Renal injury markers

Urea and creatinine were determined in the plasma using a standard colorimetry method as prescribed by the manufacturer (Randox Laboratory Ltd., UK) while renal lactate and lactate dehydrogenase (LDH) were determined using ELISA method (Aggape Diagnostic, Switzerland).

#### 2.6.3 Lipid profile

Serum and renal total cholesterol (TC), triglycerides (TG), and high-density lipoproteins (HDL) were determined using colorimetry method (Randox Laboratory Ltd., UK).

#### 2.6.4 Hematological parameters

Hematologic indices were obtained from blood samples using a hematology analyzer (KX-21 N, Sysmex Corporation, Kobe, Japan). The analyzer was accurately programmed for analysing RBC count, Hb concentration, PCV, mean corpuscular volume (MCV), and WBC count. Plasma erythropoietin was assayed using ELISA methods as described by the manufacturer (Elabscience, US).

#### 2.6.5 Oxidative stress and inflammatory markers

Renal glutathione peroxidase (GPx) was determined using a colorimetry method (Fortress Diagnostic Kit, Switzerland). Renal malondialdehyde (MDA) was estimated according to the method of Hamed et al. [28] and Akhigbe et al. [29]. Renal CAT, SOD, GSH, tumor necrotic factor-alpha (TNF-α), interleukin 6 (IL-6), interleukin 1 beta (IL-1β), interleukin 10 (IL-10), Nrf2, and NF-κB were determined using an ELISA method (Elabscience, US).

#### 2.6.6 Xanthine oxidase (XO)/Uric acid (UA) signaling

Renal XO was estimated based on the method of Afolabi et al. [30] and Akhigbe et al. [31], while UA concentration was also estimated using colorimetry method (Precision, UK).

#### 2.6.7 Statistical analysis

The test for normalcy was performed using the ‘Kolmogorov–Smirnov Test for Normality’ and GraphPad PRISM 5 software (GraphPad Software, La Jolla, California, USA) was used in the statistical analysis with a one-way analysis of variance (ANOVA) and Tukey's post hoc test. Data were reported as mean ± SD. Values of P < 0.05 were considered statistically significant.

## 3 Results

**Fig. 1:**
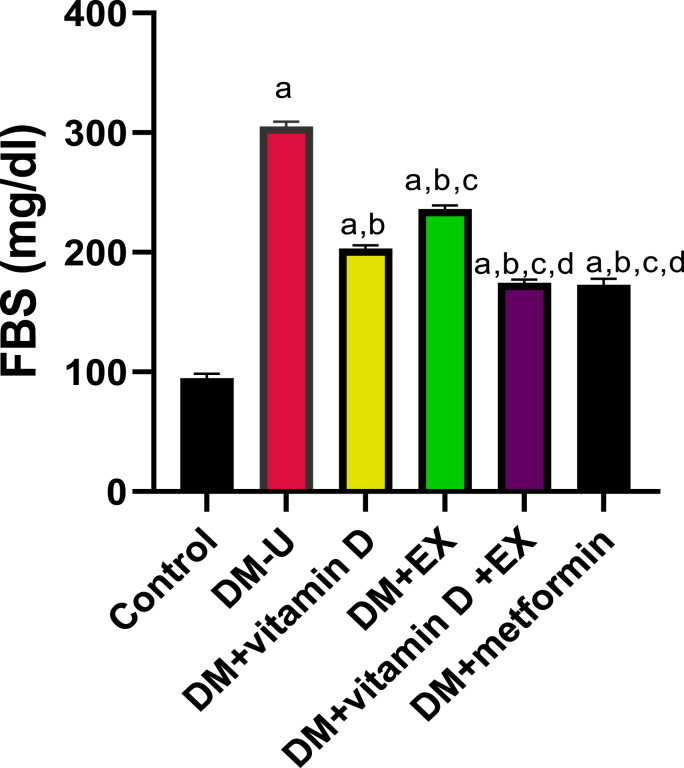
Effect of vitamin D and exercise on fasting blood sugar (FBS). ^a^P < 0.05 vs control, ^b^P < 0.05 vs DM-U; ^c^P < 0.05 vs DM + vitamin D; ^d^P < 0.05 vs DM + EX, ^e^P < 0.05 vs DM + vitamin D + EX. Data were analyzed by one way ANOVA and Tukey's posthoc test. DM-U: Diabetes untreated, EX: exercise.

FBS was significantly elevated in the diabetic untreated (DM-U) animals compared with the control animals (Fig. 1). This observed hyperglycemia was reversed in animals treated with vitamin D, exercise (EX), and co-treatment of vitamin D and EX, although, better ameliorative effect was recorded in the co-treatment group. In fact, the FBS of rats that received combination therapy is comparable with those treated with a known antidiabetic drug (metformin).

**Fig. 2:**
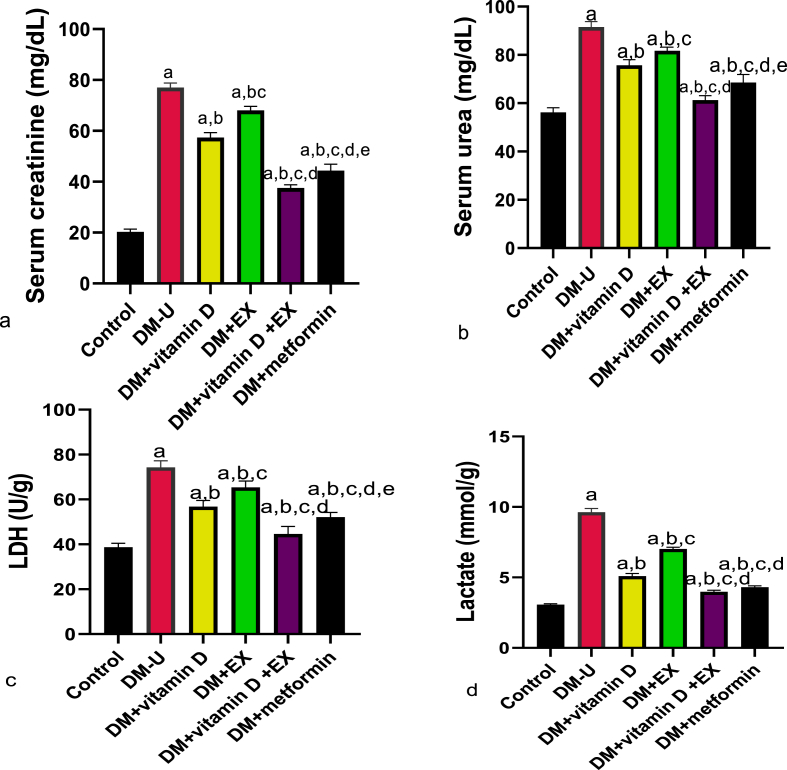
Effect of vitamin D and exercise on (a) urea (b) creatinie(c) LDH (d) Lactate. ^a^P < 0.05 vs control, ^b^P < 0.05 vs DM-U; ^c^P < 0.05 vs DM + vitamin D; ^d^P < 0.05 vs DM + EX, ^e^P < 0.05 vs DM + vitamin D + EX. Data were analyzed by one way ANOVA and Tukey's posthoc test. DM-U: Diabetes untreated, EX: exercise, LDH: lactate dehydrogenase.

Additionally, an increase in renal injury markers was observed in diabetic animals compared with the control. This observed serum urea and creatinine and renal LDH and lactate increase following diabetic induction were reversed in animals treated with vitamin D with or without EX (Fig. 2). However, vitamin D and EX co-treatment effectively reversed the observed increase in renal injury markers compared with animals treated with monotherapy.

**Fig. 3:**
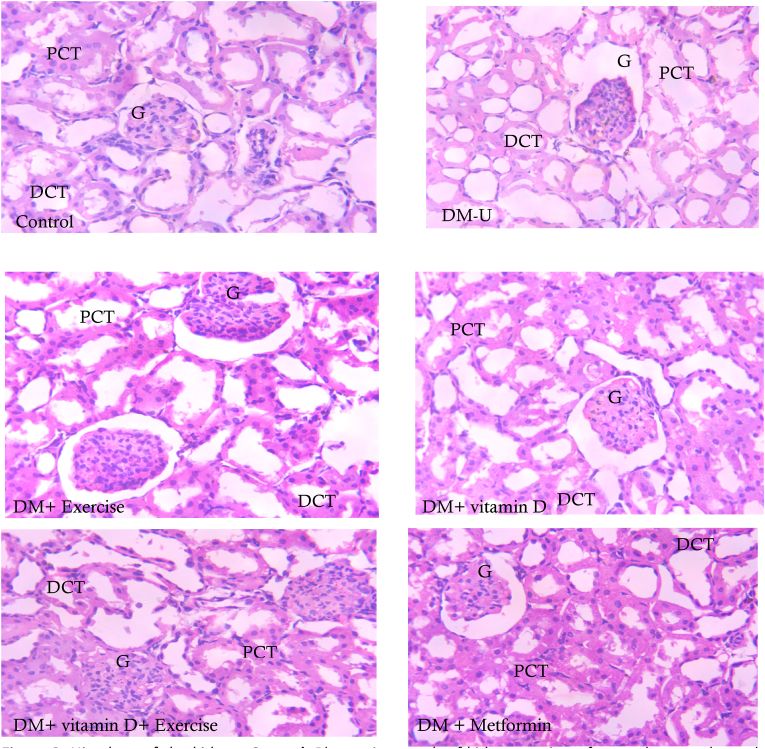
Histology of the kidney. **Control**: Photomicrograph of kidney section of control group showed normal architecture; glomerulus, proximal (PCT) and distal (DCT) convoluted tubules. **DM-U:** Major interstitial necrosis and nuclear degeneration in the PCT and DCT. **DM + Exercise:** Mild necrosis and vacuolation of the Bowman's capsule, thickened parietal layer. **DM + vitamin D:** Minor nuclear degeneration of the tubules and no evident brush borders. **DM + vitamin D + Exercise:** Mild tubular atrophic changes. **DM + Metformin:** Slightly degeneration in the epithelial cells of both proximal and distal tubules with pyknotic nuclei. Mg X 800.

**Fig. 4:**
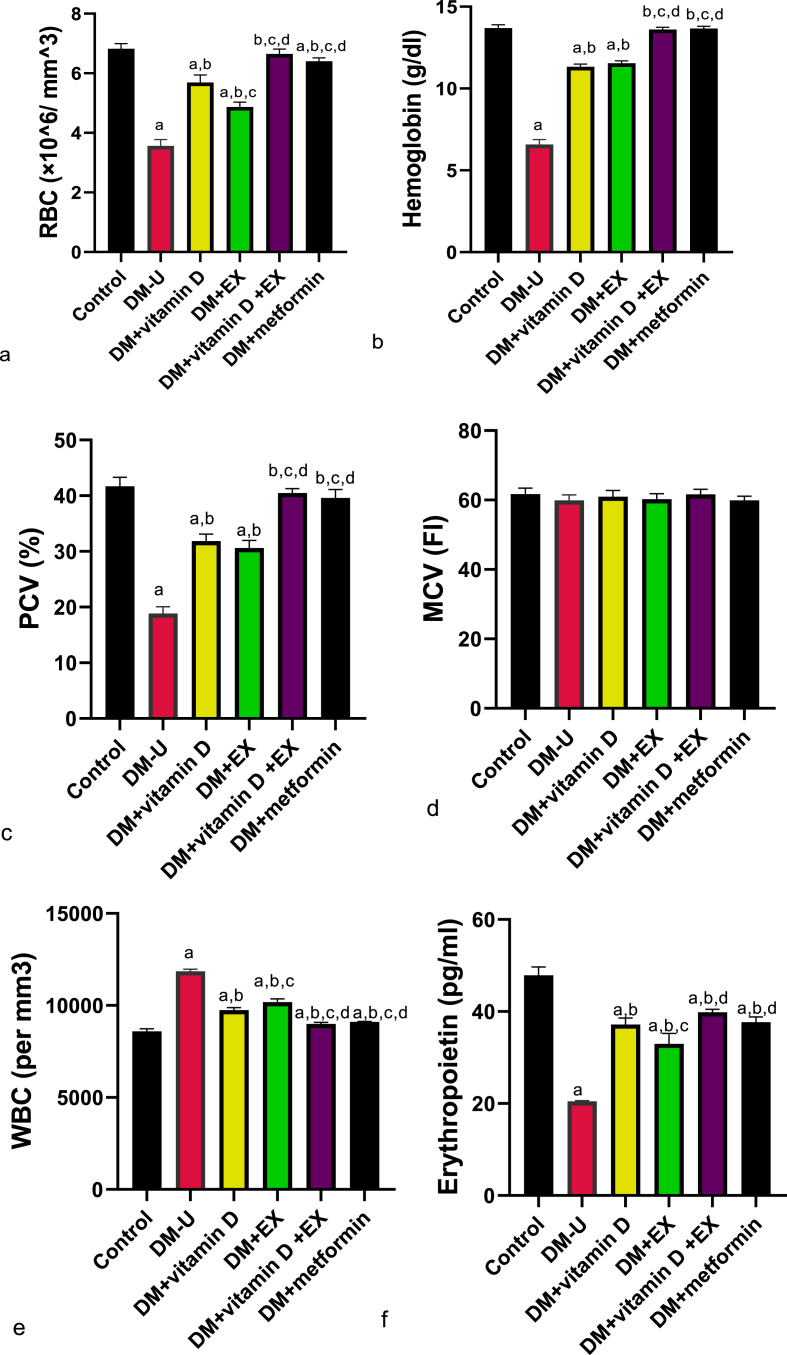
Effect of vitamin D and exercise on (a) RBC (b) hemoglobin (c) PCV (d) MCV (e) WBC. ^a^P < 0.05 vs control, ^b^P < 0.05 vs DM-U; ^c^P < 0.05 vs DM + vitamin D; ^d^P < 0.05 vs DM + EX, ^e^P < 0.05 vs DM + vitamin D + EX. Data were analyzed by one way ANOVA and Tukey's posthoc test. DM-U: Diabetes untreated, EX: exercise, RBC: red blood cells, PCV: packed cell volume, MCV: mean corpuscular volume, WBC: white blood cell. (For interpretation of the references to color in this figure legend, the reader is referred to the Web version of this article.)

As shown in Fig. 3, distortions in renal histology of animals in the DM-U were observed compared with their counterparts in the control group. Thi observed T2DM-induced distortion of normal renal histology compared with the control was ameliorated in animals treated with vitamin D and/or EX, although a better ameliorative effect was observed in animals that received vitamin D and EX co-treatment,

Furthermore, T2DM significantly reduced RBC, hemoglobin, PCV, and erythropoietin, increased WBC and insignificantly distort MCV compared with the control animals (Fig. 4). These hematological disorders were ameliorated in all the treatment groups Although no significant difference in hemoglobin and PCV of animals treated with vitamin D or physical exercise alone, the recovery rate of animals treated with vitamin D from T2DM-impaired hematological status was better than those treated with EX. Despite the effectiveness of vitamin D in reversing T2DM-induced hematological disorder, combining vitamin D with EX enhanced the recovery rate of the animals.

**Fig. 5:**
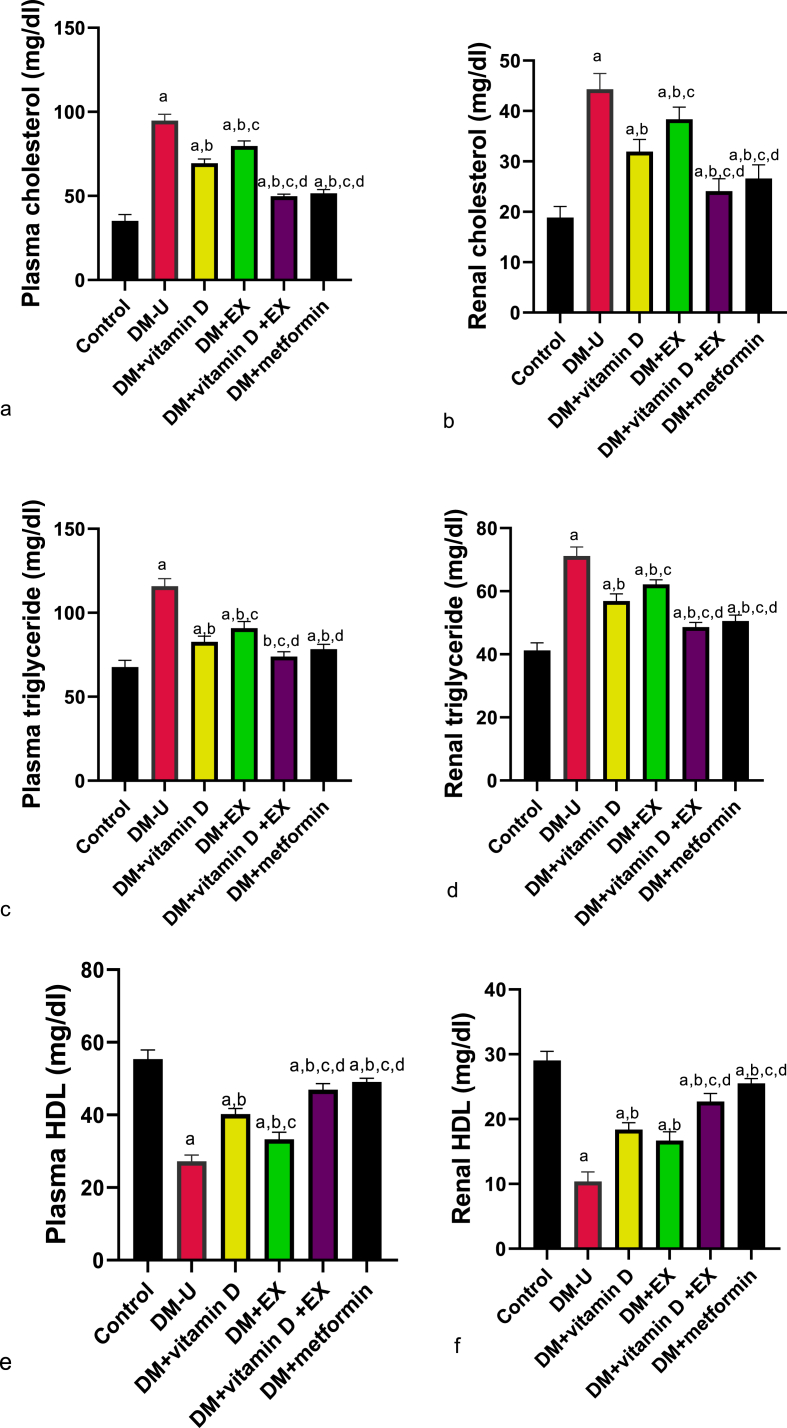
Effect of vitamin D and exercise on (a) plasma cholesterol (b) renal cholesterol (c) plasma triglyceride (d) renal triglyceride (e) plasma HDL (F) renal HDL. ^a^P < 0.05 vs control, ^b^P < 0.05 vs DM-U; ^c^P < 0.05 vs DM + vitamin D; ^d^P < 0.05 vs DM + EX, ^e^P < 0.05 vs DM + vitamin D + EX. Data were analyzed by one way ANOVA and Tukey's posthoc test. DM-U: Diabetes untreated, EX: exercise.

As shown in Fig. 5, diabetes induction significantly distorted lipid profile. This observed increase in plasma and renal cholesterol and TG and a significant decrease in HDL compared with the control were significantly reversed in all the treatment groups. Although animals that received only vitamin D treatment recovered more from T2DM-induced lipid dysmetabolism than their counterparts in the DM + EX group, better ameliorative effect was recorded in the co-treatment group.

**Fig. 6:**
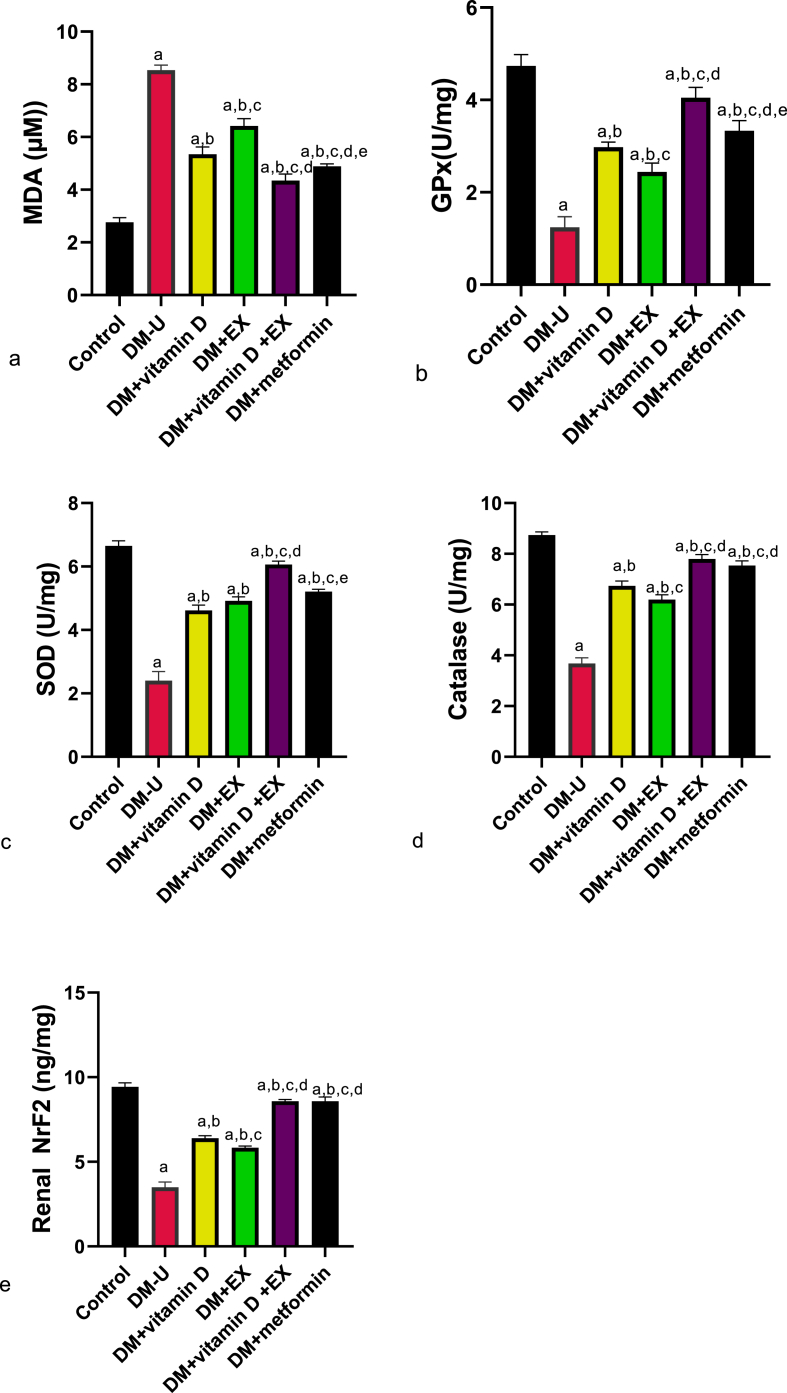
Effect of vitamin D and exercise on renal (a) MDA (b) GPx (c) SOD (d) CAT (e) Nrf2. ^a^P < 0.05 vs control, ^b^P < 0.05 vs DM-U; ^c^P < 0.05 vs DM + vitamin D; ^d^P < 0.05 vs DM + EX, ^e^P < 0.05 vs DM + vitamin D + EX. Data were analyzed by one way ANOVA and Tukey's posthoc test. DM-U: Diabetes untreated, EX: exercise, MDA: malondialdehyde, GPx: glutathione peroxidase, SOD: superoxide dismutase, Nrf2: nuclear factor erythroid-derived 2-like2.

**Fig. 7:**
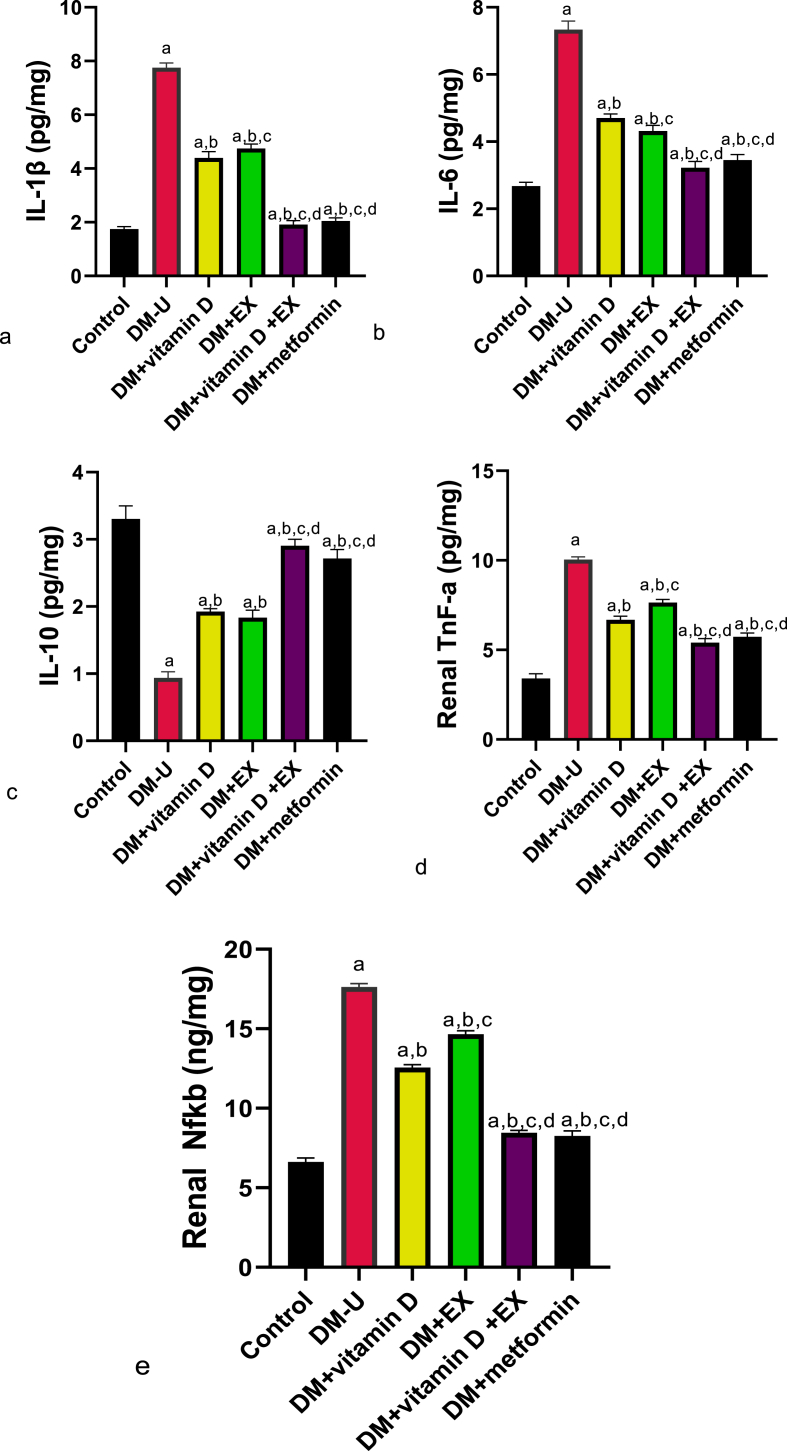
Effect of vitamin D and exercise on renal (a) IL-1β (b) IL-6 (c) IL-10 (d) Tnf-a(e) Nfkb. ^a^P < 0.05 vs control, ^b^P < 0.05 vs DM-U; ^c^P < 0.05 vs DM + vitamin D; ^d^P < 0.05 vs DM + EX, ^e^P < 0.05 vs DM + vitamin D + EX. Data were analyzed by one way ANOVA and Tukey's posthoc test. DM-U: Diabetes untreated, EX: exercise, IL-1β: Interleukin 1 beta, IL-6: Interleukin 6, IL-10: Interleukin 10, Tnf-a: Tumor necrosis factor-alpha, NfkB: Nuclear factor kappa B.

**Fig. 8:**
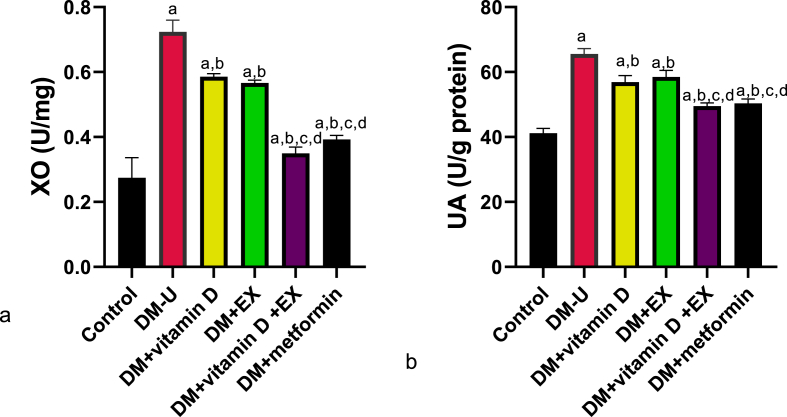
Effect of vitamin D and exercise on renal (a) XO (b) UA. ^a^P < 0.05 vs control, ^b^P < 0.05 vs DM-U; ^c^P < 0.05 vs DM + vitamin D; ^d^P < 0.05 vs DM + EX, ^e^P < 0.05 vs DM + vitamin D + EX. Data were analyzed by one way ANOVA and Tukey's posthoc test. DM-U: Diabetes untreated, EX: exercise, XO: xanthine oxidase, UA: uric acid.

Furthermore, diabetic induction significantly elevated renal MDA, IL-1β, Tnf-α, IL-6, and Nf-κB and reduced GPx, SOD, CAT, NrF2, and IL-10 compared with the control (Fig. 6, Fig. 7). These observed redox imbalance and inflammatory responses were reversed in the diabetes + vitamin D, diabetes + exercise, and diabetes + vitamin D + exercise groups. Although, animals in the diabetes + vitamin D + exercise displayed better ameliorative effects than their counterparts in the diabetes with vitamin D and diabetes with exercise groups. In the same vein, animals that received vitamin D and EX co-treatment exhibited better recovery from T2DM-induced increase in renal XO and UA compared with rats treated with a single therapy (Fig. 7) (see Fig. 8).

## 4 Discussion

This study uncovered the ameliorative roles of vitamin D supplementation and physical exercise on renal functions and integrity in T2DM Wistar rats. The results from this study established the protective roles of vitamin D supplementation and physical exercise against T2DM-induced hyperglycemia, impaired hematological markers, dysmetabolism, and oxido-inflammatory response.

Here, a low dose of STZ, in addition to HFD, was used to induce T2DM in Wistar rats. The HFD was continued for 5 weeks so that the nephrotoxic effect of STZ would not interfere with our findings since it takes about 3 weeks for the kidneys to recover from the acute mild nephrotoxic effects of STZ [32]. After the 5 weeks of continuous feeding with HFD, animals with >300 mg/dL FBS were considered diabetic and were randomly separated into different treatment groups.

Consistent with the T2DM-induced impairment in renal histoarchitecture, the observed increase in renal injury markers in diabetic untreated rats in this study are indicators of renal dysfunction. This observed nephrotoxicity was associated with impaired hematological status (evidenced by a significant decrease in RBC, Hb, and PCV and an increase in WBC count). The findings from this study that the impaired hematological status is associated with renal dysfunction can be explained by the observed decrease in erythropoietin.

Impaired renal function due to hyperglycemia-induced reactive oxygen species (ROS) has been associated with a decline in erythropoietin concentration resulting from the destruction of interstitial fibroblasts responsible for erythropoietin secretion, which usually occurs during the onset of diabetic nephropathy [33]; [6]. This renal-induced decrease in erythropoietin could lead to a decline in RBC production since erythropoietin has been shown to maintain RBC production throughout the different stages of life by preventing erythroid progenitors cell death and stimulating their development into normoblasts [34]; [35]. Additionally, the kidney has been shown to regulate hematocrit by maintaining the plasma volume and RBC mass [36]. This ability of the kidney to maintain blood volume creates a paradigm shift since it indicates that the kidney not only stimulates erythropoietin production but also regulates the hematocrit via its critmeter function. Furthermore, the observed increase in WBC and neutrophils following diabetic induction could result from T2DM-induced inflammatory response since elevated neutrophil counts constitute a significant marker for inflammation. This our hypothesis was supported by the observed increase in renal oxido-inflammatory response, and is in line with previous findings [37]; [38], that associated T2DM-induced renal dysfunction with inflammation.

Furthermore, the observed renal lipid dysmetabolism is another marker of T2DM-induced renal dysfunction since cholesterol and lipid droplet accumulation are hallmarks of diabetic kidney disease [39,40]. Lipid dysmetabolism is a major feature of DM, manifesting as dyslipidemia and ectopic lipid accumulation in organs such as kidneys [41]. Previous studies indicated that cholesterol dysmetabolism, increased lipid synthesis or uptake and fatty acid oxidation, accumulation of lipid droplets, and biologically active sphingolipids imbalance are all involved in the development and progression of T2DM-induced renal dysfunction [42]. Hence, the observed increase in plasma and renal cholesterol, triglyceride, and LDL, as well as a decrease in HDL from this study, are indicators of T2DM-induced dyslipidemia.

The observed oxido-inflammatory response from this study could stem from XO/UA signaling upregulation. XO/xanthine oxidoreductase (XOR)'s main activity is to catalyze the formation of xanthine from hypoxanthine, which will later be converted to uric acid [48]. During this process, ROS and nitric oxide (NO) are generated [44], which can lead to oxidative stress and inflammation when produced in excess. In fact, Yang et al. [43] associated the attenuation of glomerular endothelial damage and restoration of glomerular permeability following XO inhibitor administration with the oxido-inflammatory-dependent mechanism. Also, the UA produced from XO/UA signaling can act as a pro-oxidant when excessively generated. Although UA acts as an antioxidant and also supports blood pressure by regulating renal activities and hepatic metabolism [42], its excessive production has been shown to promote inflammation and oxidative stress [31]. It is noteworthy that while XO/UA signaling upregulation can impair renal functions via oxido-inflammatory response, it can also stimulate the major pathological conditions (such as hypertension, T2DM, and obesity) [42], which are the leading causes of renal dysfunction. Hence, it is reasonable to conclude that T2DM-induced renal dysfunction is at least in part associated with XO/UA upregulation.

The novelty of this study goes beyond establishing the influence of XO/UA signaling in T2DM-induced nephrotoxicity. Another significant finding from this research is the protective role of vitamin D and physical exercise in diabetic-induced nephrotoxicity. This study showed that vitamin D and EX reversed diabetic-induced impairment in circulatory erythropoietin level, renal lipid metabolism, and hematological status by modulating Nrf2/Nf-κB and XO/UA-induced renal redox imbalance and inflammatory response. This reno-protective effect of vitamin D and EX in T2DM-induced nephrotoxicity could be via ROS-inflammatory-dependent mechanisms [50] since redox imbalance has been associated with increased hemolysis [45].

Previous findings have established that antioxidant (either synthetic or natural) are good drug candidates for managing T2DM-induced redox imbalance [[46], [47], [48]]. In this study, our results showed that vitamin D is a potent antioxidant that modulates NRF-2 signaling. Nrf2 is an important endogenous antioxidant cellular resistance to oxidants. Nrf2 controls the basal and induced expression of an array of antioxidant response element–dependent genes to regulate the physiological and pathophysiological outcomes of oxidant exposure [49]. Also, Nrf2 acts as an anti-inflammatory agent by inhibiting NF-kB signaling activities [50]; [24]. Consequently, vitamin D-stimulated Nrf2 signaling reversed T2DM-induced redox imbalance and ameliorated the associated inflammatory response by inhibiting the downstream signaling involved in inflammation. Our findings that vitamin D reversed T2DM-induced redox imbalance and inflammatory response are similar to previous reports [[51], [52], [53]].

Additionally, vitamin D has been reported to ameliorate T2DM-induced ROS via XO signaling [44]. Strikingly, allopurinol (a potent XO inhibitor) has been shown to alleviate redox imbalance without inducing Nrf2 nuclear translocation [54], suggesting an independent pathway between XO/UA and Nrf2/NF-kB signaling. Hence, vitamin D prevents DKD anemia by ameliorating T2DM-induced nephrotoxicity via ROS-inflammatory-dependent mechanisms. Similar to vitamin D, EX improved T2DM-induced renal injury by attenuating redox imbalance in diabetic rats. These findings agree with previous results [55] that reported that EX maintains mitochondrial function in diabetic rats via redox homeostasis-dependent mechanisms. Interestingly, although vitamin D or EX ameliorated T2DM-induced nephrotoxicity and associated hematological disorders, they worked in synergy to provide better ameliorative effects. This is evident by the significant differences observed in animals treated with vitamin D or EX compared with animals treated with their combination.

## 5 Conclusion and future perspectives

This study indicated that physical exercise enhanced the reno-protective effect of vitamin D in T2DM-induced hematological disorders via ROS-inflammatory-dependent mechanisms. These results provide a possible insight into the protective molecular mechanisms of vitamin D and exercise against T2DM-induced DKD-associated anemia.

## 6 Limitations

Despite the convincing data presented in this study, the expression of downstream target genes for Nrf2 and NF-κB were not estimated. Future studies exploring these target genes are strongly recommended.

## Ethics approval and consent to participate

The animals used in this study were humanely catered for according to the National Institute of Health (NIH), and experimental findings were reported based on ARRIVE guidelines. Also, the guidelines stated by the National Research Council's for the Care and Use of Laboratory Animals were strictly followed and the study was approved by the institutional ethical review committee.

## Consent for publication

N/A.

## Availability of data and material

Data will be provided on request.

## Funding

None.

## CRediT authorship contribution statement

**Halimat Amin Abdulrahim:** Writing – review & editing, Visualization, Validation, Supervision, Resources, Project administration, Methodology, Investigation, Funding acquisition, Conceptualization. **Adeyemi Fatai Odetayo:** Writing – review & editing, Writing – original draft, Visualization, Validation, Supervision, Software, Resources, Project administration, Methodology, Investigation, Funding acquisition, Formal analysis, Data curation, Conceptualization. **Adeoye Tunwagun David:** Writing – review & editing, Visualization, Validation, Resources, Project administration, Methodology, Investigation, Funding acquisition. **Yusuf Funsho Abdulquadri:** Writing – review & editing, Visualization, Validation, Resources, Project administration, Methodology, Investigation, Funding acquisition. **Rofiat Oluwasheun Sheu:** Writing – review & editing, Visualization, Validation, Resources, Project administration, Methodology, Investigation, Funding acquisition. **Pelumi Kikelomo Oluwafemi:** Writing – review & editing, Visualization, Validation, Resources, Project administration, Methodology, Investigation, Funding acquisition. **Kazeem Bidemi Okesina:** Writing – review & editing, Visualization, Validation, Resources, Project administration, Methodology, Investigation. **Luqman Aribidesi Olayaki:** Writing – review & editing, Visualization, Validation, Supervision, Resources, Project administration, Methodology, Investigation, Funding acquisition, Formal analysis, Conceptualization.

## Declaration of competing interest

The authors declare that they have no known competing financial interests or personal relationships that could have appeared to influence the work reported in this paper.

## Data Availability

Data will be made available on request.
